# What Works? Prevention and Control of Sexually Transmitted Infections and Blood-Borne Viruses in Migrants from Sub-Saharan Africa, Northeast Asia and Southeast Asia Living in High-Income Countries: A Systematic Review

**DOI:** 10.3390/ijerph16071287

**Published:** 2019-04-10

**Authors:** Sajana Ghimire, Jonathan Hallett, Corie Gray, Roanna Lobo, Gemma Crawford

**Affiliations:** 1 School of Public Health, Curtin University, Kent Street, Bentley, Perth, WA 6102, Australia; 18703180@student.curtin.edu.au; 2 Collaboration for Evidence, Research and Impact in Public Health, School of Public Health, Curtin University, Kent Street, Bentley, Perth, WA 6102, Australia; J.Hallett@exchange.curtin.edu.au (J.H.); roanna.lobo@curtin.edu.au (R.L.); g.crawford@curtin.edu.au (G.C.)

**Keywords:** migrants, sexually transmitted infections, blood-borne viruses, prevention and control, systematic review

## Abstract

Migration is a significant risk factor for the acquisition of human immunodeficiency virus (HIV), hepatitis B virus (HBV) and other sexually transmitted infections (STIs). An increasing proportion of these infections in high-income countries, such as Australia, are among migrants moving from low and middle-income countries with a high prevalence of HIV, HBV and other STIs. This systematic review explored the prevention and control of HIV, HBV and other STIs in migrants (>18 years) from Southeast Asia, Northeast Asia and sub-Saharan Africa living in high-income countries with universal health care. This systematic review followed PRISMA guidelines and was registered with PROSPERO. Six academic databases were searched for articles published between 2002 and 2018. Sixteen peer-reviewed articles met the inclusion criteria, consisting of fourteen quantitative and two qualitative studies conducted in Australia, the Netherlands, Canada, Spain, Italy, and Germany. Three levels of interventions were identified: individual, community and structural interventions. Most studies addressed factors at an individual level; interventions were most commonly outreach testing for HIV, HBV and other STIs. Few studies addressed structural factors or demonstrated comprehensive evaluation of interventions. Limited population-specific findings could be determined. To prevent further transmission of HIV, HBV and other STIs, comprehensive public health approaches must consider the complex interactions between migration, health care system determinants, and broader socioeconomic and sociocultural factors.

## 1. Introduction

Migration has been an intrinsic feature of globalization and has led to increased vulnerability to a range of sexual health issues including blood-borne viruses (BBVs) and sexually transmitted infections (STIs) [1,2,3]. Over the last decade, mobility has been recognized as a risk factor for the acquisition of human immunodeficiency virus (HIV) [4,5]. An increasing proportion of STIs in high-income countries (including the US, UK, Australia and some in Europe) are diagnosed among migrant populations moving from low and middle-income countries, where there is a high prevalence of HIV and other BBVs [6,7]. An overrepresentation of notifications has been reported among migrants from sub-Saharan Africa (SSA), Northeast Asia (NEA) and Southeast Asia (SEA) regions in comparison to non-migrants in host countries [8,9,10,11].

Multiple factors influence migrants’ risk for HIV, hepatitis B virus (HBV) and other STIs and deter uptake of sexual health services. These include low levels of knowledge, the socio-economic impact of displacement and challenges in adapting to new environments and health care systems [12,13,14]. As such, migrants from some low and middle-income countries have been identified as priority groups for the prevention of HIV, HBV and other STIs in some high-income countries, including Australia [15,16].

Individual and community interventions have been the cornerstone approaches to the prevention of HIV, HBV and other STIs globally [17,18,19]. These interventions continue to be considered by the Global Strategy Report for the Prevention and Control of STIs (2016–2022) as “critically important for STI prevention, including HIV” (p. 37) [20]. Individual interventions directly focus on addressing knowledge, attitudes, skills and behaviors in a one-on-one setting, including uptake of testing, vaccination and treatment adherence [19,21]. Community interventions also address knowledge, attitudes and behaviors among high-risk populations within a community and have a strong emphasis on changing social norms [19]. These interventions include community-based education programs, mass media campaigns and peer education approaches to: reduce risk behaviors, delay the onset of first sexual intercourse, reduce the number of sexual partners, and to increase condom use [22].

However, individual and community interventions alone are insufficient to address HIV, HBV and other STIs [19,23]. The importance of structural interventions to prevent and control HIV, HBV and other STIs is recognized globally [19,20,24,25]. In addition, the meaningful involvement of at-risk communities is integral to the success of prevention, involving consultation, partnership and leadership, and working with high-risk groups to design and implement services [24,26,27]. Globally, high-income countries have seen overall success in the reduction of STIs [22]. However, in many high income countries where HIV, HBV and other STIs are exacerbated by mobility, governments have been slow to respond effectively [28].

At present, there is limited literature reporting on the prevention and control of HIV, HBV and other STIs among migrants from low and middle-income countries living in high-income countries. The aims of this review were to map interventions carried out in high-income countries, which work to prevent and control the risk of HIV, HBV and other STIs among migrant communities; identify gaps; and provide recommendations for policy, practice and research in Australia and similar contexts.

## 2. Materials and Methods

Conducted using the Preferred Reporting Items for Systematic Reviews and Meta-Analyses (PRISMA) guidelines [29,30,31], this systematic review followed consistent procedures to those used for similar reviews undertaken by the Collaboration for Evidence, Research and Impact in Public Health [6,32,33,34,35]. The review was registered in the PROSPERO International Perspective Register of Systematic Reviews to ensure the quality of implementation and reporting (Registration number: CRD42018107752).

### 2.1. Search Strategy and Information Sources

The review considered quantitative and qualitative, English language primary studies, published between 2002 and 2018 in the peer-reviewed literature. Studies included those conducted with migrants over the age of 18 years from Southeast Asia, Northeast Asia or sub-Saharan Africa living in high-income countries with universal health care. The last two criteria were applied given that the national economy and health care services of these countries arecomparable to Australia [36].

This review used the following definition of universal health coverage, a “health care system that provides healthcare and financial protection to more than 90% of the citizens of a particular country” [37]. High-income countries were those with a gross national income (GNI) per capita >$12,746 [38]. This included countries such as Australia, New Zealand, Spain and Italy [37]. A documented migrant was defined as someone “who entered a country lawfully and remains in the country in accordance with his or her admission criteria” [39] and included migrant workers, international students, refugees and asylum seekers. The review considered approaches designed to prevent and control HIV, HBV and other STIs in migrants living in high-income countries, including treatment as prevention (TasP) and interventions aimed at stigma reduction. Outcome measures included behavior change, skill development, increased testing, increased vaccination, increases in treatment uptake, increased help/health seeking and policy change. Interventions addressing hepatitis C were not included in the review as the focus was on infections that are transmitted sexually and associated risk factors.

The review excluded studies published before 2002; grey literature and non-peer-reviewed articles; studies that only included participants below 18 years of age; studies that included migrants from a high-income country moving to another high-income country; studies which did not include migrants from SEA, NEA and SSA; and studies that included non-migrants or were conducted in low-income countries.

Six databases were searched: PsycINFO, MEDLINE, ProQuest, Scopus, Global Health and Web of Science. Google Scholar was used to verify the results of the database searches. Searches were based on keyword, title and abstract. The primary researcher (SG) conducted searches for each database to ensure a full and comprehensive search was performed. Other members of the research team cross-checked the searching procedure to minimize selection bias. Reference lists from included papers were also examined to determine whether database results were exhaustive. Search dates were limited to before 30 June 2018. All applicable variations including Medical Subject Headings (MeSH) terms were used according to database specifications. Databases and search terms are listed in Table 1 below.

Retrieved journal articles were managed through Endnote X8 citation management software. After downloading articles into Endnote, duplicates were removed. The primary researcher screened the titles and abstracts and the results were reviewed by another member of the research team based on the inclusion and exclusion criteria. Where there was uncertainty regarding inclusion, other members of the research team provided input to achieve consensus. The primary researcher classified the remaining 54 relevant articles into three major categories: possibly relevant studies, background literature and excluded literature.

**Figure 1:**
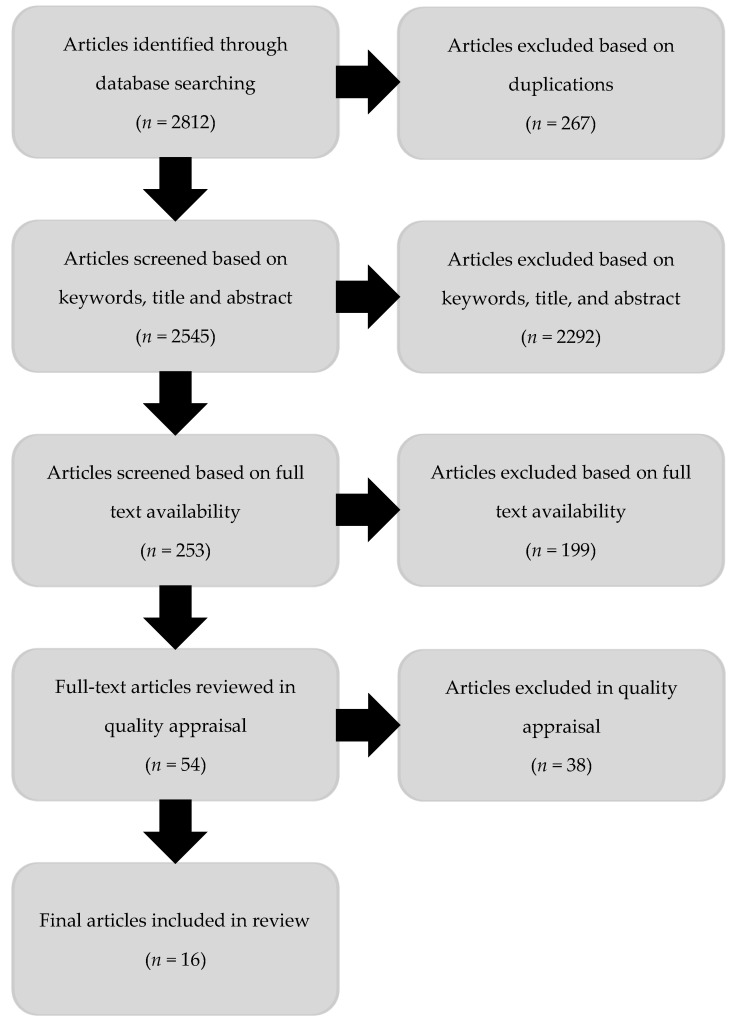
Flow diagram of review process.

The methodological quality of the included studies [40] was assessed by the primary researcher and other members of the research team using a modified checklist based on tools from the National Institute of Health and Care Excellence [41]. The criteria considered for the quality assessment checklist included: study population, methodology, outcomes and analysis. Meetings to discuss quality appraisal identified additional articles for exclusion based on the country of origin of the migrants, income and universal health care coverage of the host country. Studies focusing only on recommendations or factors associated with sexual health issues without any intervention were excluded after reviewing the full text. Figure 1 shows the process undertaken for the review.

### 2.2. Data Extraction

A standardized data extraction form was used and then the results were cross-checked by the research team for any data errors [42]. Data extraction was performed by two members of the research team. Extraction criteria included citation, participant characteristics, methods, results, key conclusions, and recommendations. The criteria were further collapsed in the final data extraction table to include study characteristics, intervention, evaluation design, sample/response and outcomes.

## 3. Results

A final sample of 16 studies met the inclusion criteria for review. The data extraction for the review is included in the Supplementary Materials (Table S1). The results have been categorized into the following domains:

- Overview of studies: location, design, methods of data collection and sample size.
- Participant characteristics: age, gender, migrants’ country of origin.
- Individual interventions: recruitment into general practice and home-based testing, provider-initiated testing and outreach testing.
- Community interventions: sexual health education and stigma reduction.
- Structural interventions: mandatory screening and changes in laws impacting on sex work.
- Study recommendations: policy, practice (clinical and health promotion) and research.

An overview of articles by intervention type is included below (Table 2). 

### 3.1. Overview of the Studies

The populations in the selected studies were migrants from SSA, NEA and SSA, living in high-income countries with universal health coverage. Of the 16 studies, four were conducted in Australia [46,52,53,54], three each in the Netherlands [44,47,50], Canada [55,57,58] and Italy [45,48,49] and one study in Spain [43], New Zealand [51] and Germany, respectively [56]. Of the 16 studies, two used qualitative methods [51,58]. The sample size of studies ranged from 15 to 46 participants in the qualitative studies and from 18 to 634,958 participants in the quantitative studies. Most studies collected information between 2002 to 2015, except for one study, where comparison was made using an earlier dataset of HIV testing in 1999 [54]. Just under half of the studies did not report ethics approval from a human research ethics committee (*n* = 7) [43,49,50,51,54,56,57].

### 3.2. Participant Characteristics

Of the 16 studies, two studies examined migrants from SSA countries [51,52]; one focused on migrants from SEA [46]; and NEA [50]; and the remaining twelve studies were among migrants from mixed regions. Fifteen studies included both male and female participants; one study was conducted with female participants only [48]. Studies varied in participants’ age with the majority including participants ≥18 years of age (*n* = 11). Three studies included participants ≥16 years [47,49,52], two studies included participants ≥15 years [56,57] and one study included participants from 14 to 21 years of age [53]. Most of the selected studies included broad migrant communities, with some studies conducted with refugees [48,51,52], culturally and linguistically diverse populations [54,55], sex-workers [43,58], asylum seekers [56]; and people living with HIV [51,55].

### 3.3. Individual Interventions

Individual interventions consisted of clinical interventions, such as testing, vaccination and treatment uptake or adherence. Of the 16 included studies, eight studies reported interventions regarding testing of STIs and uptake of HBV vaccinations [43,44,45,46,47,48,49,50]. Interventions were categorized by method used to encourage testing and vaccination.

#### 3.3.1. Recruitment into General Practice and Home-Based Testing

Three studies described attempts to recruit participants into general practice [46,54] or home-based testing [47], with limited success. Van Gemert et al. [46] conducted a pilot study in Australia with three general practice clinics to identify priority populations for HBV testing. Reviewing patient files, approximately 8% (*n* = 2674) were identified as being one of three priority population groups (Asian born or of Asian ethnicity (*n* = 2654, 99.3%), Aboriginal or Torres Strait Islanders, or people who inject drugs). A sample of priority patients (*n* = 338) received an invitation letter or phone call to attend the clinic for HBV testing. Of those, 21.6% (*n* = 73) had a subsequent consultation with a GP during the study period, and 5.5% (*n* = 4) tested for HBV, of which one patient indicated chronic HBV. The three remaining patients did not commence HBV vaccination during the intervention.

In the Netherlands, an internet-based chlamydia screening invitation was posted to all individuals aged 16–29 years old in Amsterdam, Rotterdam, and South-Limburg. In this study by Dokkum et al. [47], participants were eligible to request a home test kit, with specimens posted to the regional laboratory. It was found that reminder letters by post significantly increased requests for the testing package (*p* < 0.0001). Approximately 41% of respondents requested a testing kit after a reminder letter. Sub-Saharan African ethnicity was associated with requesting the package after a reminder letter (OR 95% CI: 1.4, 1.1–1.3).

#### 3.3.2. Provider Initiated Testing

Three studies in Italy described provider-initiated testing for migrants [45,48,49], with high levels of acceptance. Stornaiuolo et al. [45] compared two different methods of recruitment into HIV and HBV testing among migrants [45]. In the years 1999–2004, participants were actively recruited to screen for BBVs via a mobile unit in parallel with recruiting outpatients of a local migrant medical centre. In 2005–2009, only outpatients coming to the medical centre were recruited. The offer of testing had a high acceptance rate (>90%, *n* = 2681), with the majority of patients from sub-Saharan Africa (82.3%, *n* = 2202). HIV cases were significantly associated with the active recruitment process, but this was not significant for HBV. Uccella et al. [49] also reported high acceptability for a free HIV rapid test (99.2%, *n* = 825) among migrants attending an infectious disease clinic in Italy. Of migrants who tested, 71.4% were first-time testers [49].

In a screening intervention for undocumented migrant women, Frati et al. [48] found 70.9% (*n* = 537) of women agreed to be tested for human papilloma virus (HPV) and chlamydia trachomatis via a urine sample when approached at a local voluntary association providing medical and legal assistance. Women were invited to take part regardless of their reason for visiting the centre. Less than half of women who tested positive for HPV and/or *C. trachomatis* DNA agreed to undergo further investigation (Pap smear: 44.6%, 58/150, antibiotic treatment: 21.4%, 9/42).

#### 3.3.3. Outreach Testing

Targeted rapid HIV testing was conducted by Esteban-Vasallo et al. [43] in Spain and Veldhuijzen et al. [50] and Bartelsman et al. [44] in the Netherlands. In all studies, offering rapid HIV testing was effective in encouraging testing and identifying HIV cases among migrants. In Spain, outreach work was conducted with cultural mediators, mass media advertisements; and distribution of posters and brochures inviting individuals to make a free appointment at one of seven primary care services. Esteban-Vasallo et al. [43] reported that almost half (45.3%; *n* = 827) of participants were first time testers, and this was higher among migrants compared to non-migrants. In particular, being from the Indian subcontinent was significantly associated with the probability of first-time testing (82.8%, *n* = OR 16.42, 95% CI 2.08–129.88). During HIV testing week in Amsterdam, mass media, online marketing, advertising and websites provided information regarding free testing services. Almost a third of participants in the Bartelsman et al. [44] study were first-time testers, with a significantly higher percentage of first (27.2%, 61/224) and second (33.5%, 57/170) generation non-Western migrants compared to men who have sex with men (MSM) (13.2%, 40/302, *p* < 0.01).

Chronic hepatitis B testing, and an awareness campaign were conducted among Chinese first and second-generation migrants in 13 different outreach locations by Veldhuijzen et al. [50] in the Netherlands. Testing was offered free of charge as part of a larger campaign including disease awareness activities. Testing results showed half (49%; *n* = not reported) of the participants had a past or recent HBV infection, and 8.5% (*n* = 92) were diagnosed with chronic HBV infection. Of the remaining participants eligible for vaccination (51%), 22% (*n* = 124) started vaccination.

### 3.4. Community Interventions

Community interventions included mass media and group or peer education. Six of the 16 studies conducted community interventions to address sexual health knowledge and attitudes and supporting people living with HIV. Three of the studies were evaluated.

#### 3.4.1. Sexual Health Education

Of the six interventions, four studies addressed sexual health knowledge and attitudes towards safe sex [50,51,52,53]. One utilized a drama program, two studies involved peer education, and one involved community activities.

Roberts et al. [53] evaluated the ”Sharing Stories youth theatre program” with a multicultural group of 18 young people from SEA, SSA, and Middle Eastern countries. The program used drama scenarios to enable participants to recreate reality through role-play methods. The program showed increased confidence amongst young people talking about STIs among friends (59% to 69% in post-test) and increased comfort in carrying condoms (25% to 62% post-test).

Two studies involved peer education with African community members [51,52]. Drummond et al. [52] trained 10 peer educators, who conducted workshops with 58 refugees. Pre- and post-questionnaires showed a significant increase in knowledge about STIs (*p* < 0.05), mode of transmission (*p* < 0.01), condom usage for protection against STIs (*p* < 0.001), decreases in misconceptions associated with STIs and mode of transmission (*p* < 0.001), and misconceptions about protection from HIV (*p* < 0.05). The development of the New Zealand National HIV/AIDS Refugee Health Education Program by Worth et al. [51], in partnership with African refugees, utilized train-the-trainer programs to provide community educators with basic HIV information. No impact evaluation was conducted; however, 137 community educators had been trained. The program also involved the development of resources, training of health care providers, HIV support services, and relevant refugee services.

As part of a campaign reported by Veldhuijzen et al. [50] involving HBV testing, disease awareness activities were undertaken; what these involved was not reported. The evaluation showed an increase in knowledge of HBV among participants with low levels of education (40% vs. 56%, *p* = 0.06), but this was not significant.

#### 3.4.2. Mass Media Campaigns

McMahon et al. [54] conducted an ethnic media campaign (how the study was described) to increase HIV testing among culturally and linguistically diverse (CALD) communities (migrants undefined) in Australia. The campaign used “ethnic print outlets” and radio media in 14 languages to advertise free testing locations and the benefits of early HIV diagnosis. There was an increase in HIV testing among the target group in nearby clinics pre-and post-campaign (16.3% to 18.8%), however this was not significant.

#### 3.4.3. Supporting People Living with HIV

Of the 16 studies reviewed, two studies described work with people living with HIV (PLHIV) to address stigma and improve support networks [51,55]. Li et al. [55] conducted an intervention to identify challenges and effective strategies to reduce HIV stigma among diasporic communities in Canada. People living with HIV (*n* = 63) and community leaders not living with HIV (*n* = 42) were involved in acceptance commitment therapy training and social justice capacity building training to increase psychological flexibility and understand the relationship between health, equity, and social justice. The project also sought to develop advocacy and community mobilization skills. PLHIV and community leaders showed significantly decreased internalized stigma and stigma associated with HIV/AIDS respectively. PLHIV improved in readiness to be HIV champions, with increased confidence in speaking out in social situations, feeling knowledgeable, engaging others in fighting for justice and mobilizing networks after the intervention (*p* < 0.01).

The New Zealand HIV/AIDS education program was developed after in-depth interviews with 15 newly-arrived African refugees living with HIV [51]. The intervention included developing support networks for people living with HIV. No formal evaluation was conducted; however, authors reported an increase in requests for spiritual support and counseling for PLHIV and their family members.

### 3.5. Structural Interventions

Structural interventions included interventions addressing broader social, economic and political environments. Of the three studies relating to structural interventions, two studies reported on results of mandatory screening programs [56,57] and one study reported on the impact of criminalization of sex work [58].

#### 3.5.1. Criminalization of Sex Work

One of the sixteen studies described the impact of criminalization of in-call venues (locations where the client comes to the sex worker) and managers on migrant sex workers’ access to HIV and STI prevention [58]. In-depth interviews (*n* = 46) investigated the physical, social and policy environments that influenced sexual health risk, violence and access to sexual health care services in Canada. The criminalization of sex workers in in-call venues resulted in limited access to STI prevention opportunities, including restriction to condoms, and limited onsite access to sexual information and testing.

#### 3.5.2. Mandatory Screening Programs

Two studies reviewed mandatory screening programs for immigrant populations in Germany [56] and Canada [57]. Both countries have mandatory screening to identify and prevent infectious diseases, including HIV and HBV. The Federal Asylum Procedure Act in Germany requires asylum seekers to undergo a mandatory clinical examination for infectious diseases. Ackermann et al. [56] reported that of asylum seekers screened who arrived in Bavaria during the year 2015 (*n* = 95,177 for HIV screening and *n* = 94,843 for hepatitis B screening), 0.3% (*n* = not reported) were diagnosed with HIV, and 3.3% (*n* = not reported) indicated HBV. Of the HIV cases, 71.4% originated from SSA. Zencovich et al. [57], reviewed data from Canada in the year 2002–2003 of applicants for permanent settlement and temporary residents (*n* = 634,958); 932 (0.14%) new cases of HIV were identified via screening.

### 3.6. Study Recommendations

All studies made a range of recommendations for clinical practice, health education and promotion, research and policy. Recommendations generally related to improvements in clinical practices [43,44,45,46,56,57] and access to services [48,50]. Two studies [46,58] made recommendations for future research to explore the sexual health needs of migrants and the cost-effectiveness of relevant strategies. Three studies recommended implementation of health education and promotion [51,52,53] including interactive and art-based strategies, peer education, and interventions developed in partnership with the community. The remaining five studies made recommendations for the implementation of culturally appropriate interventions focusing on stigma reduction for STIs and HIV [49,51,52,54,55]. Three studies provided recommendations for policy. These included calls for the decriminalization of sex work [58] and acknowledged the effectiveness of mandatory screening in identifying infectious diseases [56,57].

## 4. Discussion

### 4.1. Overview of Findings

The purpose of this review was to identify, assess and report on interventions responding to HIV, HBV and other STIs among migrants aged 18 years or above from low and middle-income countries living in high-income countries. In summary, 16 peer-reviewed journal articles published between 2002 and 2018 met the inclusion criteria for review. This review identified three levels of interventions including individual, community, and structural interventions.

The majority of studies focused on individual interventions, including recruitment of patients into general practice and home-based testing, provider-initiated testing and outreach testing [45,46,48,54]. While the studies had mixed results, all resulted in some uptake of testing amongst the targeted sample. Testing initiated by providers at point of care showed most success, with up to 99% of patients accepting testing [45]. Recruitment into testing via mailed invitations or phone calls was low overall [46,47], although Dokkum et al. did show a significant increase in responses after reminder letters [47]. Despite its demonstrated success, provider-initiated testing presents its own challenges, particularly in general practice settings, requiring appropriate training and supportive policies [59].

Outreach testing had a high acceptance rate among migrants. Several studies reported high percentages of first-time testing among migrants, ranging from 40% to 70% [43,44,49]. Rapid testing may overcome a number of challenges to HIV testing. Two previous systematic reviews identified several barriers for migrants in accessing sexual health services, including issues around transport, eligibility, financial constraints, and lack of knowledge about sexual health issues, preventative health and health systems [34,60]. The provision of free testing at convenient locations may overcome a number of barriers to access, particularly among those who are not regularly accessing health care services [20,61].

Community interventions that included sexual health education showed short-term successes in increasing knowledge of HIV, HBV and other STIs and improving attitudes towards condoms [52,53]. In two studies, the involvement of peer educators ensured cultural-appropriateness of safe sex messaging and facilitated community ownership of the interventions [51,52]. All studies utilized traditional methods of learning, using oral communication to engage community members. No study evaluated behavior change (i.e., sexual health testing or condom use), or long-term impact. The limitations of solely education interventions in changing risk and testing behavior have been emphasized in previous studies [62,63]. This focus on individuals’ knowledge neglects to address the broader socio-cultural changes needed to create supportive environments for improved sexual health [58,64].

Only two studies addressed HIV-related stigma or support networks for PLHIV [51,55]. In one study, workshops reduced internalized stigma among PLHIV, increased confidence to talk about HIV and resulted in community mobilization. Stigma (particularly HIV-related) continues to hamper efforts in prevention and engaging people in testing, particularly among migrants where attitudes may be more negative to people living with HIV, HBV and other STIs [34,65,66]. There is a continued need to address stigma across socio-ecological levels, including individual, community and policy, to see real gains in prevention efforts [67].

In this review, structural interventions were discriminatory in nature, and seen to likely deter access to sexual health services and testing and increase stigma. The public health benefit of mandatory screening for infectious diseases, including HIV and HBV among migrants moving to high-income countries has been recognized [68,69]. However, it has been emphasized that screening should not be used as a process for exclusion for those that test positive [57,68]. Indeed, a previous systematic review noted that fear of deportation was a barrier to voluntary HIV testing for many migrants [34], particularly in the UK [70], Belgium [59], Ireland [71] and more commonly, Australia [28,61,72,73]. In countries where people living with HIV are still allowed entry, mandatory testing remains problematic. Research shows that mandatory testing results in many migrants believing that the host country is “free from HIV”, resulting in the potential for riskier sexual behavior and reduced testing [28,61]. The Joint United Nations Programme on HIV/AIDS (UNAIDS) states that mandatory testing is often delivered without pre-and post-counselling or guarantees of privacy. The UNAIDS 2016–2021 Strategy aims to see punitive laws, including mandatory HIV testing and laws against sex work, removed in line with the Sustainable Development Goal to “promote just, peaceful and inclusive societies” [74].

### 4.2. Study Design and Reporting Limitations

Most of the included studies cited a range of methodological limitations. The most frequently reported limitations were research design, data collection technique, and interpretation of the findings. Some of the included studies (*n* = 7) did not report ethical approval. Almost all the included studies (*n* = 14) were quantitative. A majority of the quantitative studies utilized self-report cross-sectional survey data. Such methods may lead to measurement error due to an overestimation of intervention effectiveness and weaken the validity of results [43,45,48,56,57]. The quasi-experimental studies conducted with pre-test post-test evaluation methods lacked randomization with no comparison made between the intervention group and control group. Such studies are prone to selection bias and reported concerns regarding internal validity [44,46,47,49,50,52,53,54,55].

The frequent limitations outlined in the studies were the use of non-probability sampling techniques; dependence on self-report measures; variations in sample size; participant drop-out rate; recall bias and self-selection bias. Few quantitative studies acknowledged the limitation of small sample sizes (fewer than 100 participants) which could have limited the power of the studies [52,55]. Only two qualitative studies were identified [51,58]. Both studies had limitations regarding sampling technique and in reporting of findings against the best practice reporting criteria [75]. Most of the studies included migrants from mixed regions of NEA, SEA and SSA and lacked information about country of birth, making it difficult to comment on the effectiveness of interventions on specific population groups.

### 4.3. Strengths and Limitations of the Review

This systematic review has several strengths. It is the first known systematic review conducted to assess the approaches designed to prevent and control HIV, HBV and other STIs in migrants from SEA, NEA and SSA living in high-income countries with universal health care systems. The use of six databases and multiple search terms across 16 years of peer-reviewed literature provided a broad scope of studies. This process has allowed an in-depth analysis of interventions conducted to address sexual health issues among migrants. This may better guide policy development, practice and research in the future. To minimize any errors and to assess the quality of the included studies, multiple researchers reviewed the database search results adopting a team approach. The scope of the review was further expanded with the inclusion of both qualitative and quantitative studies with a range of methods. The review was registered with the PROSPERO International Prospective Register of Systematic Reviews.

The study was restricted to peer-reviewed literature published in English. The grey literature and studies in languages other than English may provide valuable information to understand what interventions work with migrants. Meta-analysis and synthesis were not conducted due to the heterogeneity and high degree of variability of the included studies. The review only focused on high-income countries with universal health care systems, thus results may not be applicable to countries such as the United States. We acknowledge that low-income countries without universal health care have a wealth of information to contribute to providing additional context to the findings. The review was limited to migrants originating from SSA, SEA and NEA regions. It is likely that there are studies with migrants from other regions of birth that may have provided additional information on what interventions work with migrants. Despite these potential limitations, the study has updated and addressed a gap in the literature regarding prevention and control of HIV, HBV and other STIs among migrant populations from SEA, SSA and NEA.

### 4.4. Implications for Research, Policy, and Practice

The recommendations of the studies within this review mainly addressed issues at the individual or practice level. Emphasis was placed on methods to increase knowledge, reinforcing the effectiveness of mandatory screening, or identifying further research needs. This review showed a focus on sexual health knowledge and attitudes and screening, with limited discussion of broader policy recommendations. The majority of the studies focused on individual interventions, perhaps due to the complexity, time and resources required for interventions at the other levels. The following sections provide implications for research, policy and practice considering the broader literature.

#### 4.4.1. Research Opportunities

Studies outlined various socio-cultural barriers to the utilization of sexual health services. Further interventions are needed that consider measures beyond knowledge, and develop evaluation indicators that provide a better understanding of what works and why [76]. Such evaluation methods may include systems approaches, to generate a better understanding of contextual factors and relationships that affect outcomes, particularly for structural interventions [77]. No economic evaluations were included in this review and this is a missed opportunity to identify the cost-effectiveness of interventions [44,54].

Based on highlighted limitations regarding study design and methods within the systematic review, there is a need for studies that emphasize community involvement, such as participatory action research, co-design, or community-driven randomized control trials [27]. Reasons for low participation or high participant dropout rates were not explored and warrant further investigation in planned interventions [46]. Very few studies conducted a follow-up evaluation, and of those that did, all were within a short time-frame (less than six months) and not long enough to measure the effectiveness of the intervention longer term [49,54]. The short-term follow-up period or short interval between pre-test and post-test only showed some changes in the knowledge and attitudes of the participants, however, outcomes such as resulting changes in sexual behavior were not evaluated due to the short time frames [53,78]. Only one study involved undocumented migrants, likely due to challenges in accessing this group [48]. There were limited studies that discussed the role of treatment as prevention or linkage to support networks, particularly in relation to HIV [51].

Amongst studies included in this review, there were inconsistencies in the language used to describe migrants including migrant types (e.g., refugees, skilled migrants), country of origin and time in host country. Consistent terminology and definitions would better allow for comparison and clear understanding of risk factors and vulnerability for specific populations [79]. It is acknowledged that mobility alone is not a risk factor for HIV, HBV and other STIs [79]. Research regarding what factors increases the risk of HIV, HBV and STIs for some migrants is warranted.

#### 4.4.2. Clinical Practice Opportunities

Recommendations for clinical practice in the review focused on determining the effectiveness of screening for HIV, HBV and other STIs with targeted populations [44,56]. This includes the cost-effectiveness of different methods of offering testing (including ways of identifying high-risk groups and delivery of outreach testing) and scaling up successful pilot projects. In this review, provider initiated, and outreach testing were successful in increasing testing and identifying new cases of HIV and HBV [43,44,49,50,80]. Previous systematic reviews regarding sexual health testing of migrants have also made recommendations to increase provider-initiated and outreach testing [34,62]. While only discussed in the context of HIV for this review, the broader literature considers the availability of point of care testing (POCT) for other STIs as a priority for the management of positive cases [20].

#### 4.4.3. Health Promotion Opportunities

Studies within this review stressed the need for culturally appropriate interventions that addressed barriers to sexual health services among migrants. However, many studies targeted multiple communities without tailoring approaches that recognized heterogeneity within migrant populations and interventions were delivered without reported consultation with communities [47,48,81]. The need for active involvement of multiple stakeholders, including community leaders and religious leaders, health care providers and policymakers in designing culturally sensitive and relevant interventions for health promotion, effective service delivery and for policy change has been stressed [27,71,72]. Cultural norms, in addition to the process of resettlement, understanding of preventative health, and gender norms, influence preventative and help-seeking behavior, and as such should be considered in the design of interventions [82,83,84,85]. In addition, most interventions used a single approach (e.g., peer education), that did not address broader sociocultural factors that impact on sexual health. There is a need for multi-strategic interventions that can be sustained over longer time periods [62].

#### 4.4.4. Policy and Advocacy Opportunities

Countries need to ensure a human rights-based approach to the prevention of STIs. There have been consistent calls for the decriminalization of sex work to ensure better health, including sexual health, for sex workers [58]. The removal of mandatory screening of migrants for HIV, HBV and other STIs has been stressed by the UNAIDS and other groups [43,49,56,74].

Migration affects a broad range of social and health outcomes. The conditions of migration impact the transmission of HIV, HBV and other STIs, requiring countries to become competent in responding to population mobility [79]. Thus, more attention is needed for the development of systems which better understand and support migrants’ health, rather than increasingly restrictive migration policies [86]. Prevention efforts cannot be effective without addressing the social, economic and political determinants that influence HIV, HBV and other STI risks and vulnerability in different settings and for different populations. Continued access to prevention, treatment, care and support for HIV, HBV and other STIs is required before, during and after movement. For the most part, these factors can only be addressed through structural interventions including policy and advocacy [28,87,88].

## 5. Conclusions

Global migration has contributed to the acquisition of HIV, HBV and other STIs. An increasing proportion of these infections in high-income countries are among migrants from low and middle-income countries. Effective and coordinated responses for this priority group has been slow, with limited reporting of interventions. This study identified three main types of interventions in responding to HIV, HBV and other STIs among migrants living in high-income countries: individual, community and structural interventions. Most interventions focused on individual migrant behavior, consisting of a single strategy, with few addressing wider sociocultural factors. There is a critical need for more comprehensive interventions that consider both individual and broader socioeconomic and sociocultural factors associated with HIV, HBV and other STI testing and utilization of sexual health care services.

## Figures and Tables

**Table 1:** Search terms and databases used in the systematic review.

**Databases:**	PsycINFO, MEDLINE, ProQuest, Scopus, Global Health and Web of Science
**Migrants:**	refugee * or “international students” or “ethnic group *“ or “culturally and linguistically diverse” or “non English speaking” or “ethnic minorit *“ or “transient* and migrant *“ or immigrant * or emigrant * or “asylum seeker *“ or “migrant workers” or overseas
**Intervention:**	interven * or evaluate * or “health promot *“ or “public health” or polic * or prevent * or “best practi? e” or “good practi? e”
**Sexual Health Issues:**	“sexually transmitted disease *“ or “sexually transmitted infection *“ or “venereal disease *“ or “sexually transmissible infection *“ or “sexually transmissible disease *“ or “genital disease *“ or “human immunodeficiency virus” or “hepatitis B virus” or “blood borne virus” or STIs or HIV or STDs or BBVs

**Table 2:** Overview of articles included by intervention type.

Intervention Types	Definition and Examples	Number of Studies *(n)*	Citations
Individual	Included clinical interventions, such as testing, vaccination and treatment uptake or adherence.	8	[43,44,45,46,47,48,49,50]
Community	Included mass media and group or peer education	6	[50,51,52,53,54,55]
Structural	Included interventions addressing broader social, economic and political environments	3	[56,57,58]

## Supplementary Materials

The following are available online at https://www.mdpi.com/1660-4601/16/7/1287/s1, Table S1: Data Extraction Summary.
