# Supplementary material for: What Works? Prevention and Control of Sexually Transmitted Infections and Blood-Borne Viruses in Migrants from Sub-Saharan Africa, Northeast Asia and Southeast Asia Living in High-Income Countries: A Systematic Review
Source: Int J Environ Res Public Health. 2019 Apr 10;16(7):1287. doi: 10.3390/ijerph16071287 (PMC6480002; doi:10.3390/ijerph16071287)
Supplement: Supplementary file 1 [file ijerph-16-01287-s001.pdf]

Supplementary Materials: What works? Prevention and control of sexually transmitted infections and blood borne viruses in migrants from sub-Saharan Africa, North East Asia and South East Asia living in high-income countries: A systematic review

Sajana Ghimire, Jonathan Hallett, Corie Gray, Roanna Lobo, Gemma Crawford

Table S1. Data Extraction Summary.

Notes: CALD=culturally and linguistically diverse, CHB=chronic hepatitis B, GP = general practitioner, NA= not applicable, NR=not reported, OR=odds ratio, PLHIV=people living with HIV, SSA=sub-Saharan Africa, STI=sexually transmitted infection, TG=target group

Study Characteristics	Intervention	Evaluation Design/Methods	Sample/Response	Outcomes
**McMahon et al. (2004) [42]**<br>Ethnic media campaign on patterns of HIV testing among people from CALD communities<br>**Objectives:**<br>• Inform TGs of availability of free and anonymous HIV testing and benefits of early diagnosis;<br>• Raise awareness in TG about current HIV/AIDS issues; and<br>• Promote access by people living with HIV/AIDS from CALD backgrounds to treatment and care services.<br>**Location:** Sydney, Australia	**Community level**<br>**Educational methods**<br>**Strategies/Activities**: Printed resources in multiple languages and radio media to inform TG of availability of free HIV testing services and benefit of early diagnosis	**Length:** 1 month<br>**Design/Method:** Quasi-experimental; Comparison of number of HIV tests among TG between 1999 and 2000, and pre- and post-campaign periods in 2000 for three sexual health centers.<br>**Measures:** Number of HIV tests<br>**Participants:** CALD migrants (undefined) Comparison group: Other patients for HIV test<br>**Recruitment:**<br>**Age:** >18 years<br>**Ethics approval**: NR	**Sample:** n=1067 (pre, post campaign); n=545 (comparison).<br>**Response:**<br>99% (n= 13 dropped out)	• Increased HIV tests pre- (16.3%) to post- campaign (18.8%) though not statistically significant (p=0.31)<br>• Increase in proportion of HIV testing (10.5%, p<0.01)
**Worth et al. (2003) [43]**<br>The New Zealand HIV/AIDS refugee education program<br>**Objective:** Change attitudes and beliefs towards PLHIV and misconceptions associated with sexually transmitted infections.<br>**Location:** Auckland, New Zealand	**Community level**<br>**Educational methods**<br>**Strategies/Activities**: HIV/AIDS health promotion training and support for refugee communities; resource development; service provider training and support; and development of support networks for PLHIV.	**Length:** NR<br>**Design/Method:** Process evaluation<br>**Measures:** NR<br>**Participants:** Newly arrived male and female HIV positive African refugees.<br>**Recruitment:** Via community-based organizations.<br>**Age:** 20–35 years<br>**Ethics approval**: NR	**Sample**: n=15; 10 females and 5 males<br>**Response:**<br>100%	• Increased understanding, acceptance and active participation in project.<br>• Increased request for spiritual support and counseling for PLHIV and their family members.
**Esteban-Vasallo et al. (2014) [44]**<br>Targeted rapid HIV testing and consultation in public primary care services in Madrid<br>**Objectives:** Increase knowledge of HIV serostatus among people who belong to groups disproportionately affected by HIV.<br>**Location:** Madrid, Spain	**Individual level**<br>**Biomedical and education methods**<br>**Strategies/Activities:** Counseling and rapid HIV testing offered from seven primary care services.	**Length:** 2 years<br>**Design/Method:** Descriptive cross-sectional.<br>**Measures:** Number of HIV tests, test results and participant characteristics.<br>**Participants:** Immigrants, sex workers, heterosexual men and MSM.<br>**Recruitment:** Outreach work conducted with cultural mediators; mass media advertisement; and posters and brochures distribution.<br>**Age**: >18 years<br>**Ethics approval:** NR	**Sample:** n=1940 all study population; n=687 immigrants<br>**Response:**<br>94% (n=114 dropped out)	• HIV testing services used by large number of MSM and immigrants<br>• Higher proportion of immigrants from SEA tested for first time (p <0.05) (OR 16.42, 95% CI 2.08-129.88)<br>• Increased proportion of testing among those with no casual sexual partners (OR 1.49) and with no history of any STIs (OR 1.93).

Study Characteristics	Intervention	Evaluation Design/Methods	Sample/Response	Outcomes
**Bartelsman et al. (2017) [45]**<br>HIV testing week: lowering barriers for HIV testing among high-risk groups in Amsterdam<br>**Objectives:**<br>• Create awareness by emphasizing the importance of early testing among both professionals and inhabitants of Amsterdam, and<br>• To normalize and increase proactive HIV testing and the detection of new HIV infections.<br>**Location:** Amsterdam, Netherlands	**Individual level**<br>**Biomedical and education methods**<br>**Strategies/Activities:**<br>• Anonymous HIV rapid testing offered free of charge at various clinical and non-clinical healthcare locations<br>• Home-based testing was provided through online services	**Length**: 1 week<br>**Design/Method:** Quasi-experimental.<br>**Measures:** Number of HIV tests, HIV positivity, participant characteristics and location of test.<br>**Participants:** MSM and non-Western migrants<br>**Recruitment:** Online marketing and advertising; flyers and posters distributed at locations and via outreach; newspaper, radio and television broadcasts; and a website to provide information about testing week and locations of services.<br>**Age**: >18 years<br>**Ethics approval:** Yes	**Sample:** n=1231<br>**Response:** NA	• 32.7% received HIV test for the first time. 35% had tested more than a year before.<br>• For first- and second-generation non-Western migrants tested for the first time (27.2% and 33.5%, respectively, $p < 0.01$)
**Stornaiuolo et al. (2014) [46]**<br>Active recruitment strategy in disadvantaged immigrant populations improves the identification of HIV but not of hepatitis B or C virus infections<br>**Objectives:**<br>• Evaluate the prevalence of HIV, HBV, and HCV;<br>• Explore the factors associated with the infections; and<br>• Compare an active system of recruitment with a passive one.<br>**Location:** Caserta, Italy	**Individual level**<br>**Biomedical and education methods**<br>**Strategies/Activities:** Screening test was offered to all participants attending a mobile health unit and outpatients from health-related services and family counseling.	**Length**: 10 years<br>**Design/Method:** Cross sectional, comparison between two phases of the recruitment process.<br>**Measures:**<br>Number of HIV tests, HIV, HBV and HCV positivity and participant characteristics.<br>**Participants:** Migrants mostly from SSA and Asia<br>**Recruitment:**<br>• 1999-2004: Active recruitment through mobile unit in addition to outpatients from health-related services and family counseling<br>• 2005-2009: Recruitment via outpatients from the medical center.<br>**Age:** >18 years<br>**Ethics approval**: Yes	**Sample:** n=2681 all study population; n= 2202 SSA; n=115 Asia<br>**Response:** >90% (n=NR)	• High testing acceptance rate (>90%).<br>• No significant difference in prevalence of HBV or HCV. HIV infection significantly associated with active recruitment ($p<0.05$).<br>• For those from SSA, 8.1% were diagnosed with HBV, 2.5% with HCV and 5.4% with HIV.<br>• For those from Asia, 3.5 % were diagnosed with HCV and 1% with HIV.

**Drummond et al. (2011) [47]**<br>Using peer education to increase sexual health knowledge<br>**Objectives:** Create awareness on STIs and BBVs and address misconception about how HIV is trasmitted.<br>**Location:** Perth, Australia	**Community level**<br>**Educational methods**<br>**Strategies/Activities**: 10 peer educators undertook 9 hours of training on sexual health. Peer educators worked in pairs or groups to conduct workshops with 10-15 participants.	**Length:** NR<br>**Design/Method:** Quasi experimental, pre-test and post-test evaluation<br>**Measures:** Knowledge of STIs and HIV transmission and attitude towards condom use.<br>**Participants:** West African refugees<br>**Recruitment:** Via peer educators<br>**Age** >16 years<br>**Ethics approval**: Yes	**Sample:** n=58 post-test respondents<br>**Response:**<br>100%	• Six of seven knowledge categories increased significantly.<br>• No significant difference in attitudes towards condom use.
**Roberts et al. (2017) [48]**<br>Sharing stories youth theatre program for sexual health promotion<br>**Objectives:** Increase STI and BBV knowledge and the uptake of harm minimization strategies<br>**Setting:** Perth, Australia	**Community level**<br>**Educational methods**<br>**Strategies/Activities**: Theatre, filmmaking, art and drama used to empower communities to become peer educators to discuss sexual health education.	**Length:** 12 weeks<br>**Design/Method:** Mixed methods, pre-test and post-test evaluation.<br>**Measures:** Confidence talking about sexual health; in seeking STI testing; knowledge of where to access information and where to go for STI testing; and positive attitudes towards carrying condoms.<br>**Participants:** Young people from SEA, SSA and Middle East<br>**Recruitment:**<br>**Age:** 14 to 21 years<br>**Ethics approval**: Yes	**Sample:** n=18 pre and n=15 post<br>**Response:**<br>83% (n=3 non-attendance for post evaluation)	• Increased confidence talking about sexual health with friends and in seeking testing after unprotected sex. However, confidence talking about sexual health with family members did not increase.<br>• Increased knowledge of STIs, places to assess accurate information about sexual health and to receive sexual health services.<br>• Increase in positive attitudes towards carrying condoms and asking partners to use them.
**Zencovich et al. (2006) [49]**<br>Immigration medical screening and HIV infection for health promotion and infectious disease prevention<br>**Objectives:** Introduced mandatory HIV testing and counselling in 2001 to mitigate public health risk of HIV and for health protection of the applicant.<br>**Location:** Canada	**Structural level**<br>**Biomedical and educational methods**<br>**Strategies/Activities**: Mandatory HIV testing of permanent residency applicants.	**Length:** 2 years<br>**Design/Method:** Cross-sectional<br>**Measures:** HIV positivity and participant characteristics.<br>**Participants:** All applicants for permanent residency.<br>**Recruitment:** NA<br>**Age:** >15 years<br>**Ethics Approval**: NR	**Sample:** n=634,958<br>**Response:** NA	• 932 cases diagnosed with HIV (441 women, 491 men). Median age was 34 years.<br>• Of diagnosed cases, 67% were from Africa, 22% America, 7% from Asia and remaining 5% from other countries.<br>• 36% were refugees and 34% refugee claimants.

Study Characteristics	Intervention	Evaluation Design/Methods	Sample/Response	Outcomes
**Van Gemert et al. (2016) [50]**<br>Identification of priority populations to increase hepatitis B testing rates, 2012<br>**Objectives:** Identify people at increased risk of HBV and increase HBV testing and HBV vaccination in these populations.<br>**Location:** Melbourne, Australia	**Individual level**<br>**Biomedical and education methods**<br>**Strategies/Activities:**<br>Implementation of a system for identification of high-risk populations and a call back system to increase hepatitis B testing and vaccination where appropriate.	**Length of time:** 4 months<br>**Study design:** Pre-post intervention<br>**Measures:** HBV test and uptake of HBV vaccination.<br>**Participants:** Asian born patients, Aboriginal and/or Torres Strait Islander people and people with a history of injecting drugs who had not tested for HBV or had tested and were HBV susceptible.<br>**Recruitment:** Mailed letters and/or phone calls from clinics.<br>**Age:** ≥18 years<br>**Ethics approval:** Yes	**Sample:** n=338<br>**Response:**<br>n=4 (1%)	• 21.6% (n=73) of invited patients had a subsequent consultation with a general practitioner.<br>• Four patients tested for HBV, and one tested positive for CHB. Remaining three patients not vaccinated.
**Dokkum et al. (2012) [51]**<br>Keeping participants on board: Increasing uptake by automated respondent reminders in an internet-based Chlamydia screening<br>**Objectives:** Assess whether annual systematic, selective screening can reduce population prevalence of *Chlamydia trachomatis* (*Ct*) and prevent serious complications.<br>**Location:** The Netherlands	**Individual level**<br>**Biomedical**<br>**Strategies/Activities:** Screening procedure consisted of five steps: invitation, request of home testing kits, home sampling, sample return and checking the test result.	**Length of time:** First round April 2008, second round started from 2009.<br>**Design/Method:** Intervention<br>**Measures:** Response rate (% of package requests) and participation rate (% of sample return).<br>**Participants:** All 16-29 year-olds<br>**Recruitment:** An invitation letter, a reminder letter, two emails and an SMS.<br>**Age:** 16 to 29 years<br>**Ethics approval**: Yes	**Sample:** Round 1: n=256,400<br>Round 2: n=301,600<br>**Response:**<br>Round 1: 21% (n=52,628).<br>Round 2: 14% (n= 41,729).	• Package requests were 21% and 14% and returned samples 16% and 11.5% in round 1 and 2 respectively.<br>• 41% and 42% of participants requested the package after a reminder letter, significant for round 1 (p=0.0001). 79% and 82% returned the sample.<br>• SSA ethnicity associated with requesting the package after a reminder letter (OR (95%CI): 1.4)
**Ackerman et al. (2018) [52]**<br>Mandatory screening for infectious diseases among newly arrived asylum seekers, Bavaria, Germany, 2015.<br>**Objectives:** Assess the results of the mandatory screening procedures for HIV infection, hepatitis B and other diseases among asylum seekers.<br>**Location:** Bavaria, Germany	**Individual level**<br>**Biomedical**<br>**Strategies/Activities:** Data were extracted from the mandatory notification and laboratory information system. Demographic data captured via interviews for registration with local health authorities.	**Length of time:** Not reported<br>**Design/Method:** Cross-sectional<br>**Measures:** Serological screening of HIV and HBV<br>**Participants:** Asylum seekers undertaking HIV screening who originated from high risk countries<br>**Recruitment:**<br>**Age:** ≥15 years<br>**Ethics approval**: NR	**Sample:** n=95117<br>**Response:**<br>NA	• 0.3% tested positive for HIV. 58% were male and 15.8% female. 24.5% were in between the age group of 15-24 years. 71.4% of total cases originated from SSA<br>• 3.3 % of cases indicated HBV. Highest positivity found in asylum seekers from Sierra Leone, Senegal and Mali (17.6%, 16.2%, and 15.4%, respectively).

Study Characteristics	Intervention	Evaluation Design/Methods	Sample/Response	Outcomes
**Li et al. (2018) [53]**<br>Effects of HIV stigma reduction interventions in diasporic communities: insight from the CHAMP project<br>**Objectives:** Assess the effectiveness of two group interventions, Acceptance and Commitment Therapy/Training (ACT) and Social Justice Capacity Building (SJCB), in reducing HIV stigma and mobilizing champions to address HIV stigma.<br>**Location:** Toronto, Canada	**Community level**<br>**Educational**<br>**Strategies/Activities:** Random assignment to four half-day training sessions of ACT or ACT and SJCB.	**Length of time**: 1 year<br>**Design/Method:** Pre-post intervention<br>**Measures:** Enacted and internalized HIV stigma, and HIV champion readiness.<br>**Participants:** PLHIV and community leaders of diasporic communities.<br>**Recruitment:** Outreach to community organizations, advertisements in local (ethnic) media, and presentations in the communities.<br>**Age:** ≥18 years<br>**Ethics approval:** Yes	**Sample:** n=63 PLHIV; n=42 community leaders<br>**Response:** 63% (n=28 PLHIV and n=11 community leaders)	• Significantly decreased internalized stigma and stigma against HIV/AIDS.<br>• Speaking out in social situations, feeling knowledgeable, confident to talk, engage others to fight for justice and mobilize networks were significantly increased after the intervention ($p<0.01$)<br>• Participants reported 1090 championship activities to advocate for HIV related health equity and social justice issues.
**Frati et al. (2017) [54]**<br>A novel screening strategy for improving women's health in vulnerable populations.<br>**Objectives:** Evaluate an STI screening and measure prevalence of STIs among undocumented migrant women.<br>**Location:** Milan, Italy	**Individual level**<br>**Biomedical and educational methods**<br>**Strategies/Activities:** Implemented a counseling and preventive strategy for STIs. Collection of urine sample for the analysis of STIs.	**Length of time**: 18 months<br>**Design/Method:** Cross sectional<br>**Measures:** STI test uptake, STI positivity and participant characteristics.<br>**Participants:** Undocumented migrant women attending a migrant centre<br>**Recruitment:**<br>**Age**: ≥18 years<br>**Ethics approval:** Yes	**Sample:** n= 757<br>**Response:** 71% (n=537)	• Acceptability rate for screening among participants was high (70.9%).<br>• 24.2% indicated HPV DNA positive sample.<br>• Of total positive cases, only 43.2% agreed to undergo further investigation.
**Anderson et al. (2016) [55]**<br>Impact of criminalization of in call venues and managers on migrant sex workers access to HIV/STI prevention<br>**Objectives:** Investigate the health and safety impact of sex work laws that criminalize managers and other third-party actors who work in in-call sex work establishments.<br>**Location:** Vancouver, Canada	**Structural level**<br>**Strategies/Activities:**<br>New legislation that criminalizes sex buyers, the advertisement of sexual services and third-party actors who materially benefit in the context of a commercial enterprise was introduced in December 2014.	**Length**: Three years<br>**Design/Method:** Qualitative using key informant in-depth interview<br>**Measures:** Experiences in the sex industry, interactions with police, city officials, co-workers, managers and owners, and access to condoms, education, training and outreach services.<br>**Participants:** Asian migrant sex workers, managers and business owners of in call sex work sites.<br>**Recruitment:** Outreach to in-call venues and online.<br>**Age:** >18 years<br>**Ethics approval:** Yes	**Sample:** n=46<br>**Response:** NA	• Police and immigration raids on in-call venues and the criminalization of managers severely restrict migrant sex workers' access to condoms, health outreach services, HIV/STI testing and sexual health education.

Study Characteristics	Intervention	Evaluation Design/Methods	Sample/Response	Outcomes
**Uccella et al. (2017) [56]**<br>HIV rapid testing in the framework of an STI prevention project<br>**Objectives:**<br>• Evaluate the acceptability of HIV rapid test and estimate the percentage of newly HIV diagnoses.<br>• Evaluate knowledge, attitudes and perception about HIV and other STIs among migrants in Italy.<br>**Location:** Rome, Italy	**Individual level**<br>**Biomedical and educational methods**<br>**Strategies/Activities:** Rapid testing for HIV (free) with pre- and post-test counselling provided by trained health professionals.	**Length:** 12 months<br>**Design/Method:** Intervention with pre- and post-survey<br>**Measures:** Acceptability of HIV rapid test; number of new HIV diagnoses and knowledge, attitudes and perception of HIV/AIDS and other STIs.<br>**Participants:** Attendants at an infectious disease clinic.<br>**Recruitment:** NR<br>**Age**: 16 to 70 years<br>**Ethics approval**: NR	**Sample:** n=832<br>**Response:** 99% (n=825)<br>Pre-test survey: 46.8% (n=385)<br>Post-test survey: 15.1% (n=50)	•Acceptability rate of the HIV rapid test high. 68.7% of participants were first time testers (71.4% immigrants). 10 individuals diagnosed with HIV.<br>• 89% of participants were migrants, 19.6% and 13.6% were from Africa and Asia.<br>•Poor knowledge about HIV and STIs were found significantly associated with migrants and participants with low education levels.
**Veldhuijzen et al. (2012) [57]**<br>Identification and treatment of chronic hepatitis B in Chinese migrants.<br>**Objective:** To measure the impact of screening program for chronic hepatitis B among Chinese migrants.<br>**Location**: Rotterdam, Netherlands	**Community level**<br>**Educational**<br>**Strategies/Activities:** Disease awareness activities including free HBV testing at outreach locations. Chronic HBV referred to treatment.	**Length**: 1 year<br>**Design/Method:** Intervention with pre and post-survey.<br>**Measures:** Knowledge of HBV and prevalence of chronic HBV.<br>**Participants:** Chinese community<br>**Recruitment:** Outreach through community-based organizations.<br>**Age:** >18 years<br>**Ethics approval:** NR	**Sample:** n= 1090<br>**Response:** NA	• 8.5% diagnosed with chronic HBV infection.<br>• Level of knowledge increased significantly from 36% to 49% post-campaign among participants with low education (p=0.005).
